# Exploring Sleep Challenges and Interventions in Children with a Vision Impairment: A Scoping Review

**DOI:** 10.3390/children12121688

**Published:** 2025-12-12

**Authors:** Emma Oakes, Laura N. Cushley, Tunde Peto, Katie Curran

**Affiliations:** Centre for Public Health, Queen’s University Belfast, Belfast BT12 6BA, UK

**Keywords:** sleep, vision impairment, children

## Abstract

**Highlights:**

**What are the main findings?**

- Children with vision impairment (VI) experience a substantial burden of sleep problems, most commonly delayed sleep onset and non-24-h sleep–wake disorder.
- Evidence on interventions is limited, fragmented, and largely focused on melatonin; very few non-pharmacological or behavioural sleep interventions have been evaluated in this population.

**What are the implications of the main findings?**

- There is a pressing need for robust, well-designed studies evaluating a broader range of sleep interventions tailored to the needs of children with VI, including behavioural and environmental strategies.
- Future research should prioritise inclusive, multidisciplinary approaches that consider the developmental, psychosocial, and family impacts of sleep disturbances, supporting the design of targeted and acceptable interventions.

**Abstract:**

**Background/Objectives**: Sleep problems are highly prevalent among children with vision impairment and can negatively affect physical, emotional, and cognitive development. There is a need to identify and evaluate effective interventions in this population. This scoping review aimed to map the range of sleep challenges experienced by these children and to summarise the interventions evaluated to date. **Methods**: Systematic searches were performed in Embase, Medline, and Web of Science Core Collection. Screening was completed in Covidence, and data extraction and descriptive analysis were conducted using Microsoft Excel (version 2510) and IBM SPSS Statistics (version 30). Narrative synthesis was used to summarise findings. **Results**: Fifteen studies were included, over half of which were case reports. The vast majority (14/15) were conducted in high-income countries, leaving a significant evidence gap for low- and middle-income settings. Reported sleep challenges included delayed sleep onset, non-24-h sleep–wake disorder, early morning waking, and fragmented sleep. Interventions were predominantly pharmacological (11/15), with melatonin the most frequently evaluated. Across studies, melatonin demonstrated short-term effectiveness in improving sleep latency, duration, and parent-reported quality, though prescribing practices, dosages, and availability varied. Other pharmacological options, such as tasimelteon and vitamin B12, were rarely reported. Non-pharmacological strategies were evaluated in only a small number of studies and included behavioural interventions, structured routines, and activity-based therapies. These showed potential benefit but remain under-researched. **Conclusions**: Overall, the evidence base is small, heterogeneous, and methodologically limited. Further research is needed to develop and carefully test non-pharmacological approaches, and to compare them directly with pharmacological treatments, to provide families and clinicians with effective and sustainable options.

## 1. Introduction

Sleep plays an essential role in emotional and cognitive functioning in developing children [1]. However, children with vision impairment (VI) are disproportionately affected by sleep disturbances, primarily due to circadian rhythm disruption, with approximately 80% of children with VI being affected [2,3,4]. This problem is further exacerbated by the large population of children with VI globally, estimated to be 19 million children under 15 years of age by the World Health Organisation (WHO) [5]. In the United Kingdom (UK), an estimated 26,000 children are living with sight loss, according to the Royal National Institute for Blind People [6]. The type and severity of VI contribute to the impact that sleep problems can have. In the UK, VI severity is classified as severe sight impairment or sight impairment, based on functional vision, whereas WHO classifies VI as mild, moderate, severe, and blindness based on presenting visual acuity [7,8]. Children with VI often have additional comorbidities, including developmental disorders, neurological disorders, and mental disorders. This can cause the manifestation of sleep disorders such as non-24-h sleep–wake disorder (N24SWD) in children with complete blindness, and fragmented sleep patterns in children with partial VI [2,9]. Despite the high prevalence of sleep disturbances in this population, effective interventions remain underexplored. Addressing these problems holistically is crucial for improving overall sleep outcomes for children with VI.

Sleep disorders in this demographic are due to the important role that light perception has on maintaining a 24 h circadian rhythm, with limited and absent light perception impairing normal entrainment [3]. Circadian rhythm is controlled by the anterior hypothalamus, which houses an internal pacemaker, called the suprachiasmatic nucleus (SCN) [10]. This rhythm is synchronised using environmental cues, also known as zeitgebers, with light and dark being the most crucial of these. The SCN can regulate melatonin secretion by the pineal gland, typically occurring around 2 h before the individual’s normal sleep time, with peak melatonin secretion occurring during the middle of the night [10]. This system is regulated by norepinephrine release in response to light signals transmitted by the retinohypothalamic fibres to the SCN and relayed through the superior cervical ganglia by neural cells [11]. Melatonin release is inhibited during the day, aligning the circadian rhythm with nocturnal sleep [10,11]. In children with VI, this circadian rhythm is disrupted due to reduced light perception, contributing to sleep problems such as N24SWD. Additional sleep disorders include delayed sleep onset, frequent awakenings, and daytime sleepiness [3,9,12,13,14,15]. The Lights Out trial found that children with VI had poorer sleep quality and more nighttime wakenings compared to sighted peers [14].

VI is one of the leading causes of disabilities in children due to their reduced ability to complete cognitive tasks, associated with behaviour and attention issues [14,16]. Children with VI can often experience anxiety around bedtime or may rely on co-sleeping with a parent in order to fall asleep. Notably, a systematic review found heightened anxiety and depression in children with VI, particularly in girls [17]. From the Lights Out study, daytime sleepiness and bedtime resistance were identified to be the biggest problems for children with VI; however, this study only included four individuals with no light perception, reducing the power and limiting the generalisability of the results to the broader VI population [14]. Poor sleep in children with VI impacts both them and their parents, who often sacrifice sleep, leading to stress, conflict, and a negative effect on mental and physical health [16]. In children, sleep deprivation impairs cognitive ability, decision-making, and concentration, contributing to lower academic performance and reduced self-confidence [15,18,19]. Additionally, many children with VI are in mainstream education with a lack of specialist support, leading to lower academic achievements [20]. Further, fatigue can lead to impaired ability to socially interact and take part in extracurricular activities, exacerbated by VI [18]. These effects can be intensified by challenges faced when managing their disability [15]. This highlights the importance of further research into VI and sleep disorders, along with its effects on mental health [17].

Acknowledging the impact that poor sleep can have on children with VI, this scoping review aims to evaluate and explore interventions that show evidence of improving sleep outcomes in this population, and to identify gaps in the literature to inform future research. Important areas to be discussed include the education of sleep hygiene and the establishment of an effective, structured sleep routine [4]. A sleep-conducive environment is essential for promoting restful sleep, with factors such as sensory objects, a cool, dark room, and relaxation exercises contributing to a soothing environment [3,21]. These behavioural interventions have shown some promise in addressing the psychological impacts associated with poor sleep and VI, including anxiety and depression [17]. Additionally, light therapy may be a potential intervention for children with residual light perception; however, the extent of existing evidence remains unclear, warranting further exploration in this review [22].

This review will also examine the use of pharmacological interventions. Melatonin is the most extensively studied treatment for sleep problems among children with VI. In the United States, melatonin is commonly used because it is available over the counter [3,23,24,25,26]. Evidence from the UK suggests that melatonin can help resynchronise circadian rhythms, though in some cases treatment has been associated with phase delays [27]. Other reports have linked melatonin with an increased risk of nightmares and night-time awakenings [2]. Overall, further research is needed to evaluate the safety and effectiveness of melatonin in this population.

Given this uncertainty, a comprehensive synthesis of existing evidence is needed to clarify what is known about sleep interventions for children with VI. Accordingly, this scoping review aims to map the available literature on sleep disturbances in this population and to summarise the interventions that have been used to improve sleep, highlighting key gaps to inform future research.

## 2. Materials and Methods

### 2.1. Key Search Terms and Search Strategy

A protocol for this scoping review was registered with the Open Science Framework (OSF) and adhered to the PRISMA-ScR Checklist (Table S1), ensuring a transparent and methodologically rigorous approach [28].

Search terms were developed collaboratively by the research team, with support provided by an Information Specialist (RF) from Queen’s University Belfast (QUB) to strengthen the search process. Search concepts were iteratively refined and pre-tested to ensure comprehensive coverage of the literature and to minimise the risk of missing relevant studies.

Electronic database searches were conducted in Embase (Ovid), MEDLINE All (Ovid), and Web of Science Core Collection to collect literature on sleep problems and interventions in children with VI. In addition, Google Scholar was used to review the first 200 results, adhering to Haddaway et al.’s recommendations for comprehensive coverage [29]. This search produced only two further studies for inclusion.

The search strategy combined controlled vocabulary terms (e.g., MeSH) and free-text keywords relating to VI (including blindness and low vision), sleep disorders and sleep problems, and paediatric populations. Terms were combined using Boolean operators (AND/OR) and adapted for each database. Searches were conducted from database inception to November 2024 and were limited to human studies published in English. Full database-specific search strategies, including Boolean operators and applied limits, are provided in Supplementary Table S2.

#### 2.1.1. Inclusion Criteria

- Studies focusing on children (<18 years old) with a VI;
- Publications in the English language with no date restriction;
- Research based on sleep challenges faced by children, including interventions and management strategies to overcome them;
- Original research only.

#### 2.1.2. Exclusion Criteria

- Studies relevant to adults (age > 18 years old) with a VI;
- Studies not related to sleep challenges or interventions for children with a VI;
- Non-English publications;
- Animal studies.

### 2.2. Process of Study Selection

**Figure 1:**
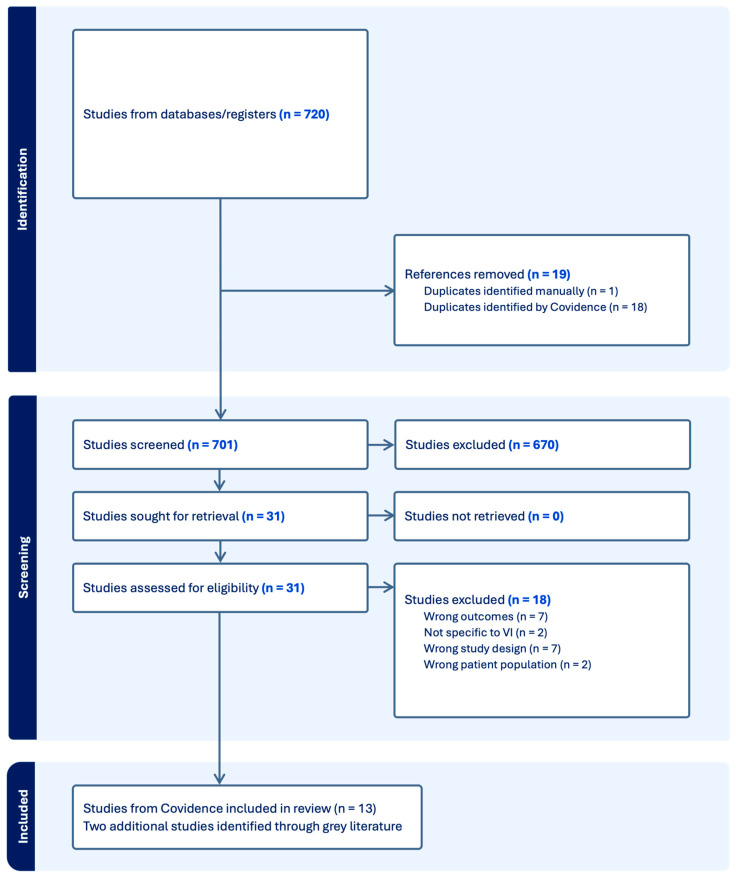
Preferred Reporting Items for Systematic Review and Meta-Analysis flow diagram of literature search and screening.

Following a search in each database, an RIS file was downloaded that included the titles, authors, and abstracts, and imported into Covidence, where screening took place. A second reviewer (KC) also performed screening. Duplicates were removed in Covidence. Following this, full texts were independently screened for eligibility based on the inclusion criteria by the first and second reviewers. Disagreements were resolved by discussion at every stage, and, when necessary, a third reviewer arbitrated (LC). A PRISMA flow diagram was developed to enhance the study selection process (Figure 1).

### 2.3. Data Collection and Management

Appropriate data on study design, sample characteristics, interventions, and outcomes were extracted, using a predefined data extraction form designed in Excel. Each extraction was completed independently by the first author (EO), and the second reviewer (KC) verified the extracted data.

### 2.4. Summarising the Evidence

Descriptive analyses were conducted in Microsoft Excel (version 2510) and IBM SPSS Statistics (version 30). Data from the included studies were analysed using a narrative synthesis approach [30]. Key themes relating to sleep problems and interventions to improve sleep in children with VI were identified and are summarised in Supplementary Table S2.

## 3. Results

### 3.1. Study Selection

A total of 720 studies were identified through database searches, with 19 duplicates removed (1 manually and 18 by Covidence), leaving 701 studies for screening (Figure 1). Of these, 670 were excluded, and 31 full-text articles were assessed for eligibility. Eighteen studies were excluded at this stage due to reasons such as wrong outcomes (n = 7), not specific to VI (n = 2), inappropriate study design (n = 7), or wrong patient population (n = 2). This resulted in 13 studies being included from Covidence. An additional two studies were identified through grey literature searches, bringing the total number of studies included in the review to 15 [3,26,30,31,32,33,34,35,36,37,38,39,40,41,42].

### 3.2. Study Characteristics

Table 1 summarises the characteristics of the 15 included studies (1991–2022). Fourteen (93.3%) were conducted in high-income countries (USA n = 3, Canada n = 2, UK n = 2, Sweden n = 2, and Switzerland, Singapore, Saudi Arabia, Japan, and the Netherlands n = 1 each), and one in Turkey, an upper–middle-income country. Study designs comprised case reports (53.3%), prospective cohorts (20%), case series (13.3%), and one each of cross-sectional, observational, and double-blind studies. Just over half (53.3%) were home-based, 40% clinical, and one was community-based. Sample sizes ranged from 1 to 100, with eight studies including a single participant. Recruitment was primarily through sleep clinics or VI programmes (80%), with two via schools for the blind and one online survey.

### 3.3. Participant Characteristics

The included studies reported a mean participant age of 8.1 years (SD 4.9), with ages ranging from 3 months to 18 years. Gender distribution was uneven across the included studies. Some cohorts consisted exclusively of either male or female participants, while others included mixed samples. One larger study reported 48.6% females, indicating a more balanced representation. However, reporting was inconsistent overall, with several studies lacking gender data and others showing an underrepresentation of females. In terms of the severity of VI, over half of the studies (53.3%) focused on children who were blind, while the remainder described varied levels, including no light perception, unspecified VI, mild to severe VI, best corrected visual acuity of 20/200 in the better eye, and blindness defined as visual acuity of 5/100 or 1/20. A small number of studies described participants with light perception only, photophobia or extreme light sensitivity, or normal light reactions (Table 2).

Across the included studies, a wide range of potential confounders were reported, with many noting neurological and developmental comorbidities. Commonly described conditions included cerebral palsy, epilepsy, moderate-to-severe intellectual disability, developmental delay, and hypoxic–ischaemic encephalopathy, with cortical VI frequently associated with more severe disability. Less common but notable conditions were partial trisomy 22, tuberculous meningitis, Ohtahara’s syndrome, leukodystrophy, autism spectrum disorder, adrenoleukodystrophy, and restless legs syndrome. Some studies also reported the use of additional treatments, such as risperidone, gabapentin, iron supplementation, and music therapy. A few studies did not describe comorbidities, while one highlighted family relocation as a contextual factor affecting follow-up. Notably, the severity of VI was not stratified, and other impairments were often excluded (Supplementary Table S3).

### 3.4. Sleep Outcome Measurement Approaches

The most frequently reported measures were sleep diaries/logs/self-reports (11 studies), followed by questionnaires (3 studies). Physiological and laboratory assessments (blood, urine, temperature) were less common, typically appearing in around three studies each. Objective monitoring tools (actigraphy, electroencephalography (EEG), respiratory parameters) and clinical/behavioural assessments (Diagnostic and Statistical Manual of Mental Disorders, Fourth Edition (DSM-IV) sleep disorder criteria, neurological evaluation, Observation of Autism and Social Interaction Difficulties (OASID), Observation of Autism and Developmental Behaviour (O-ADB)) were reported infrequently, usually in one or two studies (Table 3).

### 3.5. Overview of Sleep Interventions Evaluated Across Included Studies

Across the 15 included studies, the majority (60%) evaluated pharmacological interventions, most commonly melatonin (0.5–10 mg daily, administered in varying doses and timings, typically in the evening at or shortly before bedtime), with treatment durations ranging from 1 month to 6 years. Single studies also assessed tasimelteon (20 mg daily at 11 p.m., following melatonin failure) and methyl B12 (daily, after meals). In contrast, non-pharmacological approaches were investigated less frequently, each reported in only one study (6.7%): these included establishing a consistent bedtime routine (daily for 6 months), graduated extinction of parental attention (daily over 30 days), and a structured exercise programme (ice-skating, 1 h twice weekly for 3 months). One additional study reported on mixed strategies, incorporating both melatonin and non-pharmacological measures, though details were limited. Overall, the evidence base is heavily weighted towards melatonin, with relatively few studies exploring behavioural or lifestyle interventions (Table 4). Melatonin was the most frequently studied and commonly associated with improvements in sleep outcomes, although timing and prescribing patterns were variable. Reporting of adverse effects was inconsistent across melatonin studies. Only one case series explicitly described minor, transient side effects, while most studies did not report adverse effects (Supplementary Table S3). Tasimelteon and methyl B12 showed potential benefits, but evidence is limited to isolated studies. Behavioural and routine-based strategies demonstrated improvements in sleep and family well-being, yet remain under-investigated compared with pharmacological approaches (Supplementary Table S3).

## 4. Discussion

This scoping review demonstrates that children with VI experience a substantial burden of sleep problems, most notably delayed sleep onset and N24SWD. Previous research has similarly reported that a high proportion of children with VI experience clinically significant sleep disturbances [3]. However, despite the prevalence and impact of sleep difficulties on children and families, the available evidence addressing intervention strategies remains limited and uneven. A recent narrative review highlighted that sleep disturbance in children and young people with VI is closely linked to broader impacts on well-being and mental health, further emphasising the clinical and psychosocial importance of addressing sleep problems in this population [43].

Pharmacological interventions dominate the existing literature, with the majority of studies focusing on melatonin. Across studies, melatonin was generally effective in improving sleep parameters within days of initiation, although relapse of symptoms following treatment withdrawal was frequently reported. Importantly, evidence regarding the long-term safety of melatonin remains limited. A recent large observational study presented at the American Heart Association Scientific Sessions reported an increased risk of incident heart failure among adults with chronic insomnia who used melatonin for at least one year compared with non-users. Although this study focused on adults, was observational in design, and has not yet undergone full peer review, it raises important safety considerations regarding prolonged melatonin use. Given the widespread and often long-term use of melatonin in children with vision impairment, these findings underscore the need for caution, ongoing monitoring, and high-quality longitudinal safety studies in paediatric populations [44]. Considerable variation in prescribing practices, dosing regimens, and duration of treatment across countries reflects both clinical uncertainty and differences in healthcare systems. While melatonin is commonly introduced following unsuccessful behavioural strategies, this sequential approach has rarely been formally evaluated.

In contrast, non-pharmacological interventions were reported in only a small number of studies, predominantly case reports, and included approaches such as structured sleep–wake schedules, behavioural techniques, and sports participation. Although some improvements in sleep were observed, interpretation is limited by small sample sizes, mixed acceptability, and occasional negative psychological effects, such as reduced self-esteem. Evidence from other paediatric populations suggests that behavioural and lifestyle-based interventions can be effective, yet rigorous, VI-specific trials are lacking. Overall, pharmacological and behavioural strategies may be complementary; however, no comparative or combined intervention studies currently exist. Non-pharmacological approaches may be particularly feasible in low-resource settings, whereas melatonin use remains concentrated in high-income countries, reflecting established practice rather than clear evidence of superiority.

Recent literature published between 2021 and 2025 highlights growing scientific interest in paediatric sleep regulation, circadian rhythm disorders, and chronotherapeutic approaches; however, children with VI remain underrepresented within this expanding evidence base [3,45,46,47]. Contemporary studies examining sleep patterns in blind and visually impaired children continue to demonstrate a high burden of sleep disturbance, including delayed sleep onset and circadian disruption, using both subjective and objective measures such as actigraphy, reinforcing the clinical relevance of this issue in the present day [14,45]. Broader paediatric sleep research has advanced understanding of circadian regulation, melatonin physiology, and behavioural sleep interventions, with recent reviews and clinical frameworks emphasising chronotherapy, light-based interventions, and structured behavioural approaches as promising strategies across paediatric populations [46,48,49,50,51,52,53]. Updated literature has also documented increasing use of melatonin in children, while simultaneously highlighting uncertainty regarding long-term safety, optimal dosing, and sustained efficacy [44,51].

Despite these advances, recent studies rarely focus specifically on children with VI, and VI-specific data are often absent, limited to small subgroups, or discussed only indirectly. As a result, current recommendations for managing sleep disorders in children with VI frequently rely on extrapolation from broader paediatric or neurodevelopmental populations rather than direct evidence. Collectively, these recent publications support the conclusion that paediatric sleep and circadian research has progressed substantially in recent years, yet underline the persistent lack of rigorous, vision-specific intervention studies.

Sleep disturbance mechanisms are likely to differ according to the severity and aetiology of VI. Complete blindness is more strongly associated with circadian rhythm disorders such as N24SWD, whereas partial vision loss may be linked to fragmented sleep and behavioural sleep difficulties. These distinctions highlight the importance of tailored intervention strategies rather than a one-size-fits-all approach.

A key strength of this review is the transparent and systematic approach used throughout. A comprehensive search strategy was developed with librarian input, screening was undertaken independently by two reviewers with adjudication by a third, and the review was conducted in line with PRISMA-ScR guidelines. These steps enhanced rigour and ensured the review provided the first mapped overview of pharmacological and non-pharmacological strategies for children with VI.

Several limitations should be acknowledged. Definitions of vision impairment varied considerably across included studies, and the evidence base was characterised by small sample sizes and a predominance of case reports, with blind children disproportionately represented relative to those with partial sight. This reflects the early and fragmented nature of research in this area. A scoping review was therefore the most appropriate methodological approach to systematically map the available evidence, characterise study types, and identify key knowledge gaps, rather than to synthesise effectiveness or draw comparative conclusions. Studies were overwhelmingly conducted in high-income countries, limiting global generalisability, particularly in settings where access to sleep assessment and interventions differs substantially. Sleep outcomes were commonly assessed using subjective questionnaires prone to recall bias, with limited use of objective measures such as actigraphy. In addition, the lack of standardised sleep outcome measures hindered comparison across studies. Development of a core outcome set for sleep research in children with VI, co-produced with clinicians, families, and children, would substantially strengthen future research. Age-stratified analyses were not feasible due to inconsistent reporting and small samples; developmental implications are therefore discussed narratively rather than through subgroup analysis.

## 5. Conclusions

Children with VI experience substantial and persistent sleep difficulties, yet the existing evidence base addressing assessment and intervention remains limited, fragmented, and uneven in scope. This scoping review highlights that current research is heavily concentrated on pharmacological approaches, particularly melatonin, predominantly evaluated in high-income settings, with relatively little attention to long-term safety, combined treatment strategies, or non-pharmacological interventions.

Across studies, there is marked heterogeneity in study design, populations, and outcome measures, limiting comparison and synthesis. Evidence relating to behavioural and lifestyle-based interventions is notably sparse despite their widespread use in other paediatric populations and their potential for sustainable, low-burden implementation. Importantly, data on children with partial sight, comorbid conditions, and diverse socio-cultural contexts remain underrepresented.

Future research should prioritise multidisciplinary, multi-centre approaches that integrate ophthalmology, paediatrics, sleep medicine, psychology, and behavioural science. Key priorities include the adoption of consistent definitions of VI, integration of objective and standardised sleep outcome measures, longitudinal evaluation of safety and effectiveness, and rigorous assessment of both pharmacological and non-pharmacological interventions, alone and in combination. Embedding implementation science frameworks may further support translation of evidence-based interventions into routine clinical and community practice.

By mapping the breadth of existing evidence and clearly identifying gaps, this scoping review provides a structured foundation for the development of more effective, inclusive, and sustainable sleep interventions for children with VI and their families.

## Figures and Tables

**Table 1:** Study characteristics of the 15 included studies.

Variable Name	Number of Included Studies (n = 15)
Study location	
USA	3 (20%)
Canada	2 (13.3%)
UK	2 (13.3%)
Sweden	2 (13.3%)
Switzerland	1 (6.7%
Singapore	1 (6.7%)
Saudi Arabia	1 (6.7%)
Japan	1 (6.7%)
Netherlands	1 (6.7%)
Turkey	1 (6.7%)
Study setting	
Clinical	6 (40%)
Home-based	8 (53.3%)
Community-based	1 (6.7%)
Recruitment method	
Referral to sleep clinic/department/Visually Impaired Programmes	12 (80%)
Online survey	1 (6.7%)
School	2 (13.3%)
Type of study	
Case report	8 (53.3%)
Case series	2 (13.3%)
Prospective cohort study	3 (20%)
Observational study	1 (6.7%)
Cross-sectional survey	1 (6.7%)

**Table 2:** Participant characteristics of included studies.

Variable Name	Number of Included Studies (%) (N = 15)
Age (mean, SD) (range)	8.08 ± 4.9
	Range: 3 months to 18 years
Gender	
Exclusively female	3 (20.0%)
Exclusively male	5 (33.3%)
Mixed but male-dominant	3 (20.0%)
Mixed but female-dominant	2 (13.3%)
Balanced (50/50)	1 (6.7%)
Not reported	2 (13.3%)
Severity of VI	
Blind	8 (53.3%)
No light perception	1 (6.7%)
Not described	1 (6.7%)
Visually impaired (no description)	1 (6.7%)
VI (mild to severe)	1 (6.7%)
Best corrected vision of 20/200 in the better eye	1 (6.7%)
Visual acuity 5/100 or 1/20—blind without light perception	1 (6.7%)
No light perception, Light perception only, Photophobia or extreme light sensitivity, Normal reactions to light	1 (6.7%)

**Table 3:** Measurement of sleep outcomes.

Measurement of Sleep Outcomes (Tools/Instruments)	Number of Included Studies (%)
	(n = 15)
Questionnaires	
Sleep/behavioural questionnaires (e.g., PSQI, CSHQ, CBCL, Wiggs & Stires)	3 (20.0%)
Diaries and self/report	
Parent logs/child sleep diaries/self-reports (baseline, treatment, longitudinal monitoring)	11 (73.3%)
Physiological & Laboratory Assessments	
Blood-based measures (general blood tests, haematology, melatonin/cortisol assays)	4 (26.7%)
Urine-based measures (general urinalysis, melatonin/cortisol assays)	3 (20.0%)
Temperature monitoring (oral or body)	3 (20.0%)
Objective Sleep/Activity Monitoring	
Actigraphy	2 (13.3%)
EEG	1 (6.7%)
Respiratory parameters	1 (6.7%)
Clinical & Behavioural Assessments	
DSM-IV sleep disorder criteria	1 (6.7%)
Neurological evaluation	1 (6.7%)
OASID/O-ADB behavioural tools	1 (6.7%)

Abbreviations: Pittsburgh Sleep Quality Index (PSQI), Children’s Sleep Habits Questionnaire (CSHQ), Child Behaviour Checklist (CBCL), Wiggs & Stores Questionnaire (WSQ), Electroencephalography (EEG), Diagnostic and Statistical Manual of Mental Disorders, Fourth Edition (DSM-IV) sleep disorder criteria, Observation of Autism and Social Interaction Difficulties (OASID), Observation of Autism and Developmental Behaviour (O-ADB).

**Table 4:** Sleep interventions.

Intervention Type	Specific Intervention	Frequency/Duration	Follow-Up	Number of Studies (%) (n = 15)
Non-pharmacological	Consistent sleep routine	Daily	6 months	1 (6.7%)
	Graduated extinction of parental attention (bedtime routine, independent sleep, limited comforting of night awakenings)	Daily	30 days	1 (6.7%)
	Ice-skating	1 h, twice weekly	3 months	1 (6.7%)
Pharmacological	Melatonin (0.5–10 mg, oral, once daily; administration before bedtime	Variable dosing/timing	1 month–6 years	9 (60%)
	Tasimelteon 20 mg (oral, once daily at 11 p.m.; used after melatonin failure)	Daily	Duration not specified	1 (6.7%)
	Methyl B12 (oral, after meals)	Daily	Duration not specified	1 (6.7%)
Mixed	Survey including both melatonin and non-pharmacological interventions (details of melatonin use were not reported)	Not described	Not described	1 (6.7%)

## Data Availability

The original contributions presented in the study are included in the article/Supplementary Material, further inquiries can be directed to the corresponding author.

## Supplementary Materials

The following supporting information can be downloaded at: https://www.mdpi.com/article/10.3390/children12121688/s1, Table S1: Preferred Reporting Items for Systematic Review and Meta-Analysis Checklist; Table S2: Search terms used and number of results; Table S3: Summary of Studies on Sleep Problems and Interventions in Children with Visual Impairment.

## Abbreviations

The following abbreviations are used in this manuscript:

VI	Vision impairment
WHO	World Health Organization
SCN	Suprachiasmatic nucleus
N24SWD	Non-24-h sleep–wake disorder
QUB	Queen’s University Belfast
PSQI	Pittsburgh Sleep Quality Index
CSHQ	Children’s Sleep Habits Questionnaire
CBCL	Child Behaviour Checklist
WSQ	Wiggs & Stores Questionnaire
DSM-IV	Diagnostic and Statistical Manual of Mental Disorders, Fourth Edition
EEG	Electroencephalography
OASID	Observation of Autism and Social Interaction Difficulties
O-ADB	Observation of Autism and Developmental Behaviour

## References

[1] Vriend J., Davidson F., Rusak B. (2015). Emotional and Cognitive Impact of Sleep Restriction in Children. Sleep Med. Clin..

[2] Lockley S.W., Arendt J., Skene D.J. (2007). Visual impairment and circadian rhythm disorders. Dialogues Clin. Neurosci..

[3] Ingram D.G., Cruz J.M., Stahl E.D., Carr N.M., Lind L.J., Keirns C.C. (2022). Sleep Challenges and Interventions in Children with Visual Impairment. J. Pediatr. Ophthalmol. Strabismus.

[4] Khan S.A., Heussler H., McGuire T., Dakin C., Pache D., Norris R., Cooper D., Charles B. (2011). Therapeutic Options in the Management of Sleep Disorders in Visually Impaired Children: A Systematic Review. Clin. Ther..

[5] World Health Organization (2023). Blindness and Visual Impairment. https://www.who.int/news-room/fact-sheets/detail/blindness-and-visual-impairment.

[6] Key Statistics About Sight Loss, RNIB. https://www.rnib.org.uk/professionals/research-and-data/key-information-and-statistics-on-sight-loss-in-the-uk/.

[7] Leissner J., Coenen M., Froehlich S., Loyola D., Cieza A. (2014). What explains health in persons with visual impairment?. Health Qual. Life Outcomes.

[8] The Criteria for Certification, RNIB. https://www.rnib.org.uk/your-eyes/navigating-sight-loss/registering-as-sight-impaired/the-criteria-for-certification/.

[9] Mindell J.A., Marco C.M. (1997). Sleep Problems of Young Blind Children. J. Vis. Impair. Blind..

[10] Zee P.C., Attarian H., Videnovic A. (2013). Circadian Rhythm Abnormalities. Lifelong Learn. Neurol..

[11] Claustrat B., Brun J., Chazot G. (2005). The basic physiology and pathophysiology of melatonin. Sleep Med. Rev..

[12] Wee R., Gelder R.N. (2004). Sleep disturbances in young subjects with visual dysfunction. Ophthalmology.

[13] Tabandeh H., Lockley S.W., Buttery R., Skene D.J., Defrance R., Arendt J., Bird A.C. (1998). Disturbance of Sleep in Blindness. Am. J. Ophthalmol..

[14] Hayton J., Marshall J., Dimitriou D. (2021). Lights out: Examining sleep in children with vision impairment. Brain Sci..

[15] Guner N., Hayton J.A. (2024). Parental and Child Sleep: Children with Vision Impairment, Autistic Children, and Children with Comorbid Vision Impairment and Autism. Brain Sci..

[16] El-Sheikh M., Kelly R.J. (2017). Family Functioning and Children’s Sleep. Child Dev. Perspect..

[17] Augestad L.B. (2017). Mental Health Among Children and Young Adults with Visual Impairments: A Systematic Review. J. Vis. Impair. Blind..

[18] Tadić V., Pring L., Dale N. (2009). Attentional processes in young children with congenital visual impairment. Br. J. Dev. Psychol..

[19] Ashworth A., Hill C.M., Karmiloff-Smith A. (2015). The Importance of Sleep: Attentional Problems in School-Aged Children with Down Syndrome and Williams Syndrome. Behav. Sleep Med..

[20] Education Service Provision for Children with Vision Impairment. https://www.rnib.org.uk/documents/677/Full_report_-_Educational_Provision_Great_Britain_Report.doc.

[21] Mindell J.A., Williamson A.A. (2018). Benefits of a bedtime routine in young children: Sleep, development, and beyond. Sleep Med. Rev..

[22] van Maanen A., Meijer A.M., van der Heijden K.B., Oort F.J. (2016). The effects of light therapy on sleep problems: A systematic review and meta-analysis. Sleep Med. Rev..

[23] Poza J.J., Pujol M., Ortega-Albás J.J. (2022). Melatonin in sleep disorders. Neurología.

[24] Waldron A.Y., Spark M.J., Dennis C.M. (2016). The Use of Melatonin by Children: Parents’ Perspectives. J. Clin. Sleep Med..

[25] Jan J.E., O’Donnell M.E. (1996). Use of melatonin in the treatment of paediatric sleep disorders. J. Pineal Res..

[26] Palm L., Blennow G., Wetterberg L. (1997). Long term melatonin treatment in blind children and young adults with circadian sleep-wake disturbances. Dev. Med. Child Neurol..

[27] Skene D.J., Arendt J. (2007). Circadian rhythm sleep disorders in the blind and their treatment with melatonin. Sleep Med..

[28] Tricco A.C., Lillie E., Zarin W. (2018). PRISMA Extension for Scoping Reviews (PRISMA-ScR): Checklist and Explanation. Ann. Intern. Med..

[29] Haddaway N.R., Woodcock P., Macura B. (2015). Making literature reviews more reliable through application of lessons from systematic reviews. Conserv. Biol..

[30] Lapierre O., Dumont M. (1995). Melatonin Treatment of a Non-24-hour Cycle in a Blind Retarded Child Sleep-Wake. Biol. Psychiatry.

[31] Jan J.E., Espezel H., Appleion R.E. (1994). The treatment of sleep disorders with melatonin. Dev. Med. Child Neurol..

[32] Espezel H., Jan J.E., O’Donnell M.E., Milner R.A. (1996). The use of melatonin to treat sleep-wake-rhythm disorders in children who are visually impaired. J. Vis. Impair. Blind..

[33] Palm L., Blennow G., Wetterberg L. (1991). Correction of non–24-h sleep/wake cycle by melatonin in a blind retarded boy. Ann. Neurol..

[34] Ross C., Davies P., Whitehouse W. (2002). Melatonin treatment for sleep disorders in children with neurodevelopmental disorders: An observational study. Dev. Med. Child Neurol..

[35] Tzischinsky O., Pal I., Epstein R., Dagan Y., Lavie P. (1992). The importance of timing in melatonin administration in a blind man. J. Pineal Res..

[36] Tubbs A.S., Grandner M.A., Combs D. (2019). Refractory Insomnia in an Adolescent with Total Blindness. Yale J. Biol. Med..

[37] Suhumaran S., Yeleswarapu S.P., Daniel L.M. (2020). Congenital blindness and autism spectrum disorder (ASD): Diagnostic challenges and intervention options. BMJ Case Rep..

[38] Jan M.M.S. (2000). Melatonin for the Treatment of Handicapped Children with Severe Sleep Disorders. Pediatr. Neurol..

[39] Mindell J.A., Goldberg R., Fry J.M. (1996). Treatment of a Circadian Rhythm Disturbance in a 2-Year-Old Blind Child. J. Vis. Impair. Blind..

[40] Tomoda A., Miike T., Matsukura M. (1995). Circadian rhythm abnormalities in adrenoleukodystrophy and methyl B12 treatment. Brain Dev..

[41] Dursun O.B., Erhan S.E., Ibiş E.Ö., Esin I.S., Keleş S., Şirinkan A., Yörük Ö., Acar E., Beyhun N.E. (2016). The effect of ice skating on psychological well-being and sleep quality of children with visual or hearing impairment. Disabil. Rehabil..

[42] Vervloed M.P.J., Hoevenaars E., Maas A. (2003). Behavioral Treatment of Sleep Problems in a Child with a Visual Impairment. J. Vis. Impair. Blind..

[43] Canavan R.F., Hayton J., Tibber M.S., Dekker T.M., Wood L.A.G., Crossland M.D. (2025). Well-being, mental health and sleep in children and young people with vision impairment: A narrative review. Prog. Brain Res..

[44] American Heart Association (2025). Long-Term Use of Melatonin Supplements to Support Sleep May Have Negative Health Effects. AHA Newsroom. https://newsroom.heart.org/news/long-term-use-of-melatonin-supplements-to-support-sleep-may-have-negative-health-effects.

[45] Adhikari S., van Nispen R.M.A., Poudel M., van Rens F., Elsman E.B.M., van der Werf Y.D., van Rens G.H.M.B. (2023). Sleep Patterns in Children with Blindness: A Comparison with Normally Sighted Peers. Investig. Ophthalmol. Vis. Sci..

[46] Giannotta G., Ruggiero M., Trabacca A. (2024). Chronobiology in Paediatric Neurological and Neuropsychiatric Disorders: Harmonizing Care with Biological Clocks. J. Clin. Med..

[47] Spruyt K. (2025). The Power of Pediatric Sleep: Shaping Healthy Evidence-Based 24/7 Lifestyles for Generation Alpha. Pediatr. Discov..

[48] Händel M.N., Andersen H.K., Ussing A., Virring A., Jennum P., Debes N.M., Laursen T., Baandrup L., Gade C., Dettmann J. (2023). The short-term and long-term adverse effects of melatonin treatment in children and adolescents: A systematic review and GRADE assessment. eClinicalMedicine.

[49] Owens J., Simakajornboon N., Kotagal S., Gringras P., Melatonin Task Force, International Pediatric Sleep Association (IPSA) Practice and Policy Committee, IPSA Board of Directors (2025). Melatonin use in managing insomnia in typically developing (TD) children: A technical report. Sleep Med..

[50] Meneo D., Gavriloff D., Cerolini S., Baldi E., Schlarb A., Nobili L., Baglioni C. (2025). A Closer Look at Paediatric Sleep: Sleep Health and Sleep Behavioural Disorders in Children and Adolescents. J. Sleep Res..

[51] Owens J., Simakajornboon N., Kotagal S., Gringras P., Melatonin Task Force, International Pediatric Sleep Association (IPSA) Practice and Policy Committee, IPSA Board of Directors (2025). Melatonin use in typically developing (TD) children: International Pediatric Sleep Association (IPSA) Expert Consensus Recommendations for Healthcare Providers. Sleep Med..

[52] Kok E.Y., Kaur S., Mohd Shukri N.H., Abdul Razak N., Takahashi M., Teoh S.C., Tay J.E.F., Shibata S. (2024). The role of light exposure in infant circadian rhythm establishment: A scoping review perspective. Eur. J. Pediatr..

[53] Flynn L., Peltz J., Perreault M., Lieberman L.J., Beach P. (2025). The Relationship Between Sleep and Physical Activity for Children with Visual Impairments: A Scoping Review. J. Vis. Impair. Blind..

