# Supplementary material for: Exploring Sleep Challenges and Interventions in Children with a Vision Impairment: A Scoping Review
Source: Children (Basel). 2025 Dec 12;12(12):1688. doi: 10.3390/children12121688 (PMC12731822; doi:10.3390/children12121688)
Supplement: Supplementary file 1 [file children-12-01688-s001.zip › children-3988746-supplementary.pdf]

Supplementary Material

Table S1: *Preferred Reporting Items for Systematic Review and Meta-Analysis Checklist*

SECTION	ITEM	PRISMA-ScR CHECKLIST ITEM	REPORTED ON PAGE #
**TITLE**			
Title	1	Identify the report as a scoping review.	1
**ABSTRACT**			
Structured summary	2	Provide a structured summary that includes (as applicable): background, objectives, eligibility criteria, sources of evidence, charting methods, results, and conclusions that relate to the review questions and objectives.	2
**INTRODUCTION**			
Rationale	3	Describe the rationale for the review in the context of what is already known. Explain why the review questions/objectives lend themselves to a scoping review approach.	3-4
Objectives	4	Provide an explicit statement of the questions and objectives being addressed with reference to their key elements (e.g., population or participants, concepts, and context) or other relevant key elements used to conceptualize the review questions and/or objectives.	3-4
**METHODS**			
Protocol and registration	5	Indicate whether a review protocol exists; state if and where it can be accessed (e.g., a Web address); and if available, provide registration information, including the registration number.	5
Eligibility criteria	6	Specify characteristics of the sources of evidence used as eligibility criteria (e.g., years considered, language, and publication status), and provide a rationale.	5-6
Information sources*	7	Describe all information sources in the search (e.g., databases with dates of coverage and contact with authors to identify additional sources), as well as the date the most recent search was executed.	5-6
Search	8	Present the full electronic search strategy for at least 1 database, including any limits used, such that it could be repeated.	18
Selection of sources of evidence†	9	State the process for selecting sources of evidence (i.e., screening and eligibility) included in the scoping review.	5-6
Data charting process‡	10	Describe the methods of charting data from the included sources of evidence (e.g., calibrated forms or forms that have been tested by the team before their use, and whether data charting was done independently or in duplicate) and any processes for obtaining and confirming data from investigators.	5-6
Data items	11	List and define all variables for which data were sought and any assumptions and simplifications made.	10,32

SECTION	ITEM	PRISMA-ScR CHECKLIST ITEM	REPORTED ON PAGE #
Critical appraisal of individual sources of evidence§	12	If done, provide a rationale for conducting a critical appraisal of included sources of evidence; describe the methods used and how this information was used in any data synthesis (if appropriate).	N/A
Synthesis of results	13	Describe the methods of handling and summarizing the data that were charted.	N/A
**RESULTS**			
Selection of sources of evidence	14	Give numbers of sources of evidence screened, assessed for eligibility, and included in the review, with reasons for exclusions at each stage, ideally using a flow diagram.	7
Characteristics of sources of evidence	15	For each source of evidence, present characteristics for which data were charted and provide the citations.	9
Critical appraisal within sources of evidence	16	If done, present data on critical appraisal of included sources of evidence (see item 12).	N/A
Results of individual sources of evidence	17	For each included source of evidence, present the relevant data that were charted that relate to the review questions and objectives.	9-13
Synthesis of results	18	Summarize and/or present the charting results as they relate to the review questions and objectives.	9-13
**DISCUSSION**			
Summary of evidence	19	Summarize the main results (including an overview of concepts, themes, and types of evidence available), link to the review questions and objectives, and consider the relevance to key groups.	13
Limitations	20	Discuss the limitations of the scoping review process.	14
Conclusions	21	Provide a general interpretation of the results with respect to the review questions and objectives, as well as potential implications and/or next steps.	14
**FUNDING**			
Funding	22	Describe sources of funding for the included sources of evidence, as well as sources of funding for the scoping review. Describe the role of the funders of the scoping review.	N/A

Table S2*: Search terms used and number of results*

**Searches**		
**Embase**<br>(1974 – 2024 November 19)	**Medline All**<br>(1946 – November 19, 2024)	**Web of Science**<br>(All fields, no date restriction)
1. exp visual disorder/<br>2. exp visual impairment/<br>3. exp blindness/<br>4. exp sleep disorder/<br>5. insomnia/<br>6. wakefulness/<br>7. circadian rhythm sleep disorder/ or circadian rhythm disorder/ or dyssomnia/ or advanced sleep phase syndrome/ or delayed sleep phase syndrome/ or irregular sleep-wake rhythm disorder/ or non-24-hour sleep-wake disorder/<br>8. sleep apnea syndromes/<br>9. sleep apnoea.mp.<br>10. intervention*.mp.<br>11. treatment*.mp.<br>12. exp phototherapy/<br>13. strateg*.mp.<br>14. sleep hygiene/<br>15. support group/<br>16. 1 or 2 or 3<br>17. 4 or 5 or 6 or 7 or 8 or 9<br>18. 10 or 11 or 12 or 13 or 14 or 15<br>19. 16 and 17 and 18<br>20. limit 19 to child <unspecified age><br>21. limit 20 to (human and english language)<br>22. conference abstract/<br>23. 21 not 22<br><br>**Total: 645**	1. exp Vision Disorders/<br>2. Visually Impaired Persons/ or vis* impair*.mp.<br>3. Visual Acuity/ or light perception.mp.<br>4. Blindness/<br>5. exp Sleep Wake Disorders/<br>6. insomnia.mp.<br>7. Wakefulness/<br>8. Sleep/ or circadian rhythm disorder*.mp. or Sleep Disorders, Circadian Rhythm/<br>9. exp Sleep Apnea Syndromes/<br>10. sleep apnoea.mp.<br>11. intervention*.mp.<br>12. treatment*.mp.<br>13. exp Phototherapy/<br>14. support group.mp.<br>15. strateg*.mp.<br>16. Sleep Hygiene/<br>17. 1 or 2 or 3 or 4<br>18. 5 or 6 or 7 or 8 or 9 or 10<br>19. 11 or 12 or 13 or 14 or 15 or 16<br>20. 17 and 18 and 19<br>21. limit 20 to "all child (0 to 18 years)"<br>22. limit 21 to (english language and humans)<br><br>**Total: 51**	1. "vis* impair*" or blindness or "low vision" or "sight impair*" or "light perception" or "registered blind"<br>2. "sleep disorder*" or insomnia or "sleep quality" or "circadian rhythm disorder*"<br>3. intervention* or treatment* or "light therap*" or cbt or "sleep routine*"<br>4. child* or infant* or adolescent* or teenager* or paediatric* or toddler*<br><br>**Total: 24**

Table S3*: Summary of Studies on Sleep Problems and Interventions in Children with Visual Impairment*

Author, Year	Country	Design	Sample (n, age range)	Severity of VI	Sleep problem(s)	Intervention	Main outcomes	Measures	Potential Confounders
**Dursun et al., 2014 [41]**	Turkey	Prospective cohort	40 (8–16y)	VI (20/200 or worse)	Poor sleep quality (PSQI>6)	Ice skating (1h, twice weekly, 3 months)	Improved sleep & behaviour; decreased self-concept/peer relations	Questionnaires: PSQI; SDQ; PHCSCS (pre/post)	Other impairments largely excluded; no VI-severity stratification
**Espezel et al., 1996 [32]**	Canada	Prospective observational cohort	100 (< 1 year –17y)	VI (mild–severe)	Onset delay, night/day reversal, free running	Melatonin 2.5–10mg. Timing: Not consistently specified; melatonin given in the evening, timing individualised based on sleep-wake pattern. Adverse effects: Not reported.	Improved sleep; VI with neuro comorbidities	Sleep charting (parent logs); melatonin estimations not completed	Neurological disorders common; CVI associated with more severe disability
**Ingram et al., 2022 (Survey) [3]**	USA	Cross-sectional survey	72 (1–16y)	Mixed (NLP–normal light response)	Initiation, maintenance, nightmares, napping	Mixed strategies (routine, melatonin, co-sleeping, relaxation, etc.)	Melatonin & consistent routines most effective; VI children had worse sleep	Questionnaires: CSHQ and open-ended survey items	Developmental delay and neurological disorders reported
**Jan et al., 1994 [31]**	UK	Case series	9 (3–14y)	Blind	Fragmented sleep, delayed onset	Melatonin 2–10mg. Timing: administered in the evening, typically 30-60 mins before bedtime. Adverse effects: Minor, transient effects reported in some children (e.g., morning drowsiness). Generally well tolerated.	Improved sleep; minor adverse events	Sleep charting/recording; haematology & urinalysis at 3 and 6 months	Mental impairment, cerebral palsy, epilepsy
**Jan, 2000 [38]**	Saudi Arabia	Cohort	7 (1–11y)	NR	CRSWD, fragmented sleep	Melatonin 3mg. Timing; evening dosing, aligned with desired sleep onset (timing not standardised).	Effective in regulating sleep	DSM-IV sleep-disorder criteria; parent logs; neurologist evaluation	Epilepsy, cerebral palsy, moderate–severe mental impairment, migration disorder, Hypoxic–Ischaemic Encephalopathy

						Adverse effects: Not reported.		(hours sleep/awakenings/delayed onset/early arousals)	
**Lapierre & Dumont, 1995 [30]**	Canada	Case report	1 (5y)	NLP	N24SWD	Melatonin 0.5mg. Timing; administered in the evening timed to entrain circadian rhythm. Adverse effects: Not reported.	Entrainment to 24h cycle; increased mood/learning	Sleep diaries; blood tests	Partial trisomy 22; mental & psychomotor impairment
**Mindell et al., 1996 [39]**	USA	Case report	1 (2y)	Blind	Onset delay, night waking, day sleep	Behavioural schedule (consistent sleep routine)	Improved sleep; decreased night waking	Sleep diary entries (22-week monitoring; 6-month review)	None described
**Palm et al., 1991 [33]**	Sweden	Case report	1 (9y)	Blind	N24SWD	Melatonin 0.5mg ± placebo. Timing: melatonin given before bedtime. Adverse effects: Not reported.	Improved rhythm with melatonin	EEG; 24-h oral temperature; urine melatonin & cortisol (RIA)	Severe mental impairment; epilepsy
**Palm et al., 1997 [26]**	Sweden	Case series	5 (3–16y)	Blind	N24SWD (average day length 24.4–25.5h)	Melatonin 0.5–4mg. Timing: evening dosing. Adverse effects: Not reported.	Improved rhythm; 1 relapsed to pretreatment rhythm	Sleep diaries (pre/post); body temperature; blood/urine melatonin	Severe–moderate mental impairment; one patient deaf
**Ross et al., 2002 [34]**	UK	Observational	6 (1–7y)	VI (not detailed)	Onset delay, night waking, fragmented sleep	Melatonin 2.5–10mg. Timing: evening dosing; escalation to maximum tolerated dose. Adverse effects: Not reported.	5 VI children benefited	Parent sleep diaries (baseline week; treatment week at max dose)	Comorbidities including CP, Tuberculous meningitis, PEHO-like syndrome, Ohtahara's syndrome, leukodystrophy
**Suhumaran et al., 2020 [37]**	Singapore	Case report	1 (6y)	Blind	Sleep disturbances	Melatonin (dose NR). Timing: not reported. Adverse effects: Not reported.	Improved sleep & behaviour	Multidisciplinary reassessment; OASID/O-ADB tools	Autism spectrum disorder; risperidone; music therapy; structured schedule

**Tomoda et al., 1995 [40]**	Japan	Case report	1 (13y)	Blind	N24SWD	Methyl B12 (1500µg daily)	Normalised sleep; increased nocturnal melatonin secretion	Diary log; blood melatonin/cortisol/β-endorphin; chronograph	Adrenoleukodystrophy
**Tubbs et al., 2019 [36]**	USA	Case report	1 (17y)	Blind	N24SWD	Melatonin (ineffective) so Tasimelteon 20mg tested. Timing: Evening. Adverse effects: Melatonin ineffective rather than poorly tolerated; no adverse effects reported.	Improved N24; family-reported sleep gains	Actigraphy; respiratory parameters; caregiver reports	Restless legs syndrome; gabapentin (100 mg nightly) & iron replacement
**Tzischinsky et al., 1992 [35]**	Switzerland	Case report (double-blind)	1 (18y)	Blind	Delayed sleep phase	Melatonin 5–10mg vs placebo. Timing: Best effect when administered 2-3 hours before bedtime. Adverse effects: None reported.	Best timing = 2–3 h before bedtime; small advance and increased duration	Actigraphy; oral temperature; self-report; 6-month follow-up	None described
**Vervloed et al., 2003 [42]**	Netherlands	Case report	1 (4.5y)	Visual acuity 5/100 or 1/20 - blind without light perception	Initiation, night waking, nightmares, daytime sleepiness	Graduated extinction behavioural programme (30 days)	Improved sleep; parents reported increased obedience/cheerfulness	Questionnaires: CBCL; sleep diary; Wiggs & Stores sleep questionnaire	Family moved to a new city between intervention and 3-month follow-up

Abbreviations: VI= visual impairment; NLP= no light perception; RLS= restless legs syndrome; NR=not reported; PSQI=Pittsburgh Sleep Quality Index; SDQ=Strengths and Difficulties Questionnaire; PHCSCS=Piers–Harris Children's Self-Concept Scale; CSHQ=Children's Sleep Habits Questionnaire; DSM-IV=Diagnostic and Statistical Manual of Mental Disorders, 4th Edition; EEG=Electroencephalogram; RIA= Radioimmunoassay; OASID=Observation of Autism in people with Sensory and Intellectual Disabilities; O-ADB=Observation of Autism in people with Developmental Disabilities; CBCL=Child behaviour Checklist; CVI=Cerebral Visual Impairment; CP=Cerebral Palsy; PEHO=Progressive Encephalopathy with Edema, Hypsarrhythmia, and Optic Atrophy
